# The Effect of Organic Solid Waste Compost on Soil Properties, Growth, and Yield of Swiss Chard Crop (*Beta vulgaris* L.)

**DOI:** 10.1155/2023/6175746

**Published:** 2023-10-23

**Authors:** Temesgen Kebede, Dereje Diriba, Ararsa Boki

**Affiliations:** ^1^Department of Natural Resource Management, Dilla University, P.O. Box 419, Dilla, Ethiopia; ^2^Department of Chemistry, Dilla University, P.O. Box 419, Dilla, Ethiopia; ^3^Department of Plant Science, Dilla University, P.O. Box 419, Dilla, Ethiopia

## Abstract

In Dilla town and the university compound, huge amounts of biodegradable solid waste (BDSW), which include food and farm and yard wastes, are generated from student and staff cafeterias and animal farms. Improper treatment and disposal of this waste resulted in contamination of surface water and soil, air pollution, and spreading of diseases. On the other hand, soil fertility of most arable lands of Dilla Zuria woreda is highly depleted due to low levels of soil fertility management practices and inorganic-based farming. These factors make a considerable contribution to the degradation of fertile soil and reduction of yield. Thus, the management of BDSW through composting is important to minimize environmental problems and improve the soil fertility of arable lands. However, the effects of BSWC compost on plant growth and crop yield are highly variable in different studies. This study aimed to evaluate the effect of food waste compost (FWC) and leaf yard compost (LYC) at different application rates on the soil properties, growth, and yield responses of Swiss chard (*Beta vulgaris* L.). Food waste, leaf and yard waste, and animal dung were collected and composted in a heap-composting method. The produced organic amendments were applied to soil at an application rate of 5, 10, and 15 t/ha, respectively. Two harvesting times were considered, and at each leaf harvesting time, plant growth parameters (height, leaf number, leaf area, and fresh weight) were analyzed; after the second harvesting time, soil properties were analyzed. Results indicated that increasing rates of FWC significantly (*p* < 0.001) increased the plant height, leaf area, and fresh yield of Swiss chard. The application of 15 t/ha of FWC also increased SOC, TN, available P, and CEC. Food waste was recycled through composting as a soil amendment to improve soil properties and the yield of Swiss chard.

## 1. Introduction

Every year in the world, about 1.7–1.9 billion metric tons of municipal solid waste are generated from offices, markets, and industries [1]. Biodegradable solid waste (BSW) is a nonliquid waste material that can be decomposed by bacteria or other natural organisms and cannot contribute to pollution. These wastes are the result of activities in homes, farms, businesses, and industries. BSW, which includes food waste, leaf yard waste, animal manure, etc., generated from agricultural lands and municipal areas, causes large-scale pollution of land and water [2]. The deposition of biodegradable waste results in pollution of the environment, greenhouse gas emissions, and transboundary migration of organic micropollutants and volatile heavy metals [3].

In Dilla town and university compounds, huge amounts of food and farm and yard wastes are generated from student and staff cafeterias and agriculture farms and deposited without any management system. Improper treatment and disposal of this waste resulted in contamination of surface water and soil, air pollution, and spreading of diseases [4]. Intensive cultivation of soils, low levels of soil fertility management practices, and inorganic-based farming agriculture for the production of cereal and horticultural crops in the study area resulted in a reduction of soil fertility, mainly due to a decrease in organic matter and nutrients [5]. Thus, organic-based agriculture is important to minimize deposition and environmental problems associated with open dumping of biodegradable solid waste into compost to improve soil fertility and crop yield.

Composting BSW converts potentially toxic materials into a steady state, which can improve soil quality for plant growth [6]. Composting technology has become an effective management approach for converting biodegradable organic waste into useful compost to provide soil nutrients, improve water-holding capacity, improve aeration, and increase crop production [7]. Composting biodegradable waste improves soil fertility and reduces the incidence of pathogenic microorganisms [8, 9]. Composting biodegradable waste is also important to reduce environmental impacts [10]. Moreover, BSW compost is a sustainable solution to improve the quantity and quality of agricultural products [11] and the chemical properties (N, P, and K) of soil [12–14]. Composting BSW also increases microbial activity and microbial biomass associated with symbiotic root associations [15]. Therefore, BSW compost application is an alternative to reduce the negative effects of commercial fertilizer, enhance agricultural sustainability, and improve soil aggregation [16, 17].

Several studies have been conducted to examine the effect of BSW compost on cereals, legumes [18, 19], and vegetables [20, 21]. However, composting types of biodegradable waste, doses, stages, and forms of use are highly varied among study area farmers. There have also been very few studies evaluating the types and application rates of food waste, leaf, and yard compost on the physical, chemical, and yield properties of Swiss chard (*Beta vulgaris* L.).

Therefore, this study aimed to investigate the effect of application rates of FWC and LYC on selected soil physicochemical properties and yields of Swiss chard (*Beta vulgaris* L.).

## 2. Materials and Methods

### 2.1. Description of the Study Area

The experiment was carried out at the Botanical Garden and Ecotourism Center Nursery Site at Dilla University, Gedeo zone, Ethiopia. It is geographically located at 6° 27′ 05″ north latitude and 38° 30′ 36″ east longitudes, with an altitude of 1466 m.a.s.l., and is located 376 m.a.s.l. south of Addis Ababa.

The study site has an annual rainfall of 1001–1800 mm and a temperature range of 12–25°C [5]. It also has suitable agroecology for vegetables, coffee, enset, and other fruits. There was also potential for animal production. In this study, Swiss chard (*Beta vulgaris* L.) was used as a test crop. It is a leafy vegetable and is extremely consumed by people who are living in Gedeo zone [5].

### 2.2. Compost Preparation

Food waste compost (FWC) is any inedible part of food removed from the food supply chain to be composted with animal manure and water with the help of microorganisms. Leaf yard waste compost (LYC) is defined as compost prepared from leaves and other organic materials, such as grass clippings, with animal manure and water with the help of microorganisms. Food waste (onion, potato, and carrot peel and cabbage inedible parts) was collected from Dilla University students' cafeterias, local markets, and households. Leaf and yard waste, as well as animal manure, was collected from agricultural farms. The nonbiodegradable fraction was manually separated from the organic fraction, which was then shredded and composted in a heap method. The waste mixtures were composted in heaps in the size of 1.5 m high, 2 m wide, and 2 m long. This size is advantageous to turn the heap, improve the supply of oxygen, and prepare a large amount of compost [22]. Heap temperature was checked every ten days, and it ranged from 34 to 60°C. It was the ideal temperature for heap composting [23]. During the maturation phase, the heaps were turned on a regular and weekly basis to improve the O_2_ level within the heap. Heap moisture was kept under control by adding enough water to keep the moisture content at or above 50%. By increasing the population of aerobic microorganisms, this aeration speeds up the composting process.

### 2.3. Seedling Preparation and Field Management

Seeds of yellow Swiss chard were obtained from the Research Center of the Ethiopian Institute of Agricultural Research Center. Swiss chard seeds were raised on a well-prepared seed bed. The seeds were drilled in rows with ten-centimeter row spacing and covered with dried grass. Weeding, watering, and protection of seedlings from insects and fungal diseases were carried out in order to produce healthy seedlings.

Before transplanting seedlings into the experiment field, the land was plowed by the hand, the bed was leveled, smoothed, and loosened uniformly, and then one week before transplanting seedlings, compost was mixed on the surface of plots with rakes to bare soil. It was attempted to maintain a uniform distribution of compost over the surface during application, and it was further mixed to an approximate soil depth of 10 cm. Finally, strong and healthy Swiss chard seedlings were transplanted in the experimental field with a spacing of 30 cm between plants and 45 cm between rows. A total of 21 plots, each measuring 3 m × 1.8 m (5.4 m^2^), were used for the experiment. Management of planted seedlings (watering, weeding, and protection) was practiced until the second harvesting period.

### 2.4. Experimental Design and Treatments

During growing seasons, the experiment was conducted using a randomized complete block design (RCBD) consisting of two types of compost: food waste (FWC) and leaf and yard waste (LYC) in the presence of animal manure with a ratio of 3 : 1 by using three application rates (5, 10, and 15 tons/ha) with three replications. The experiment was designed with twenty-one plots having *T*1 = control (no compost application); *T*2 = 5 t/ha (2.7 kg); FWC, *T*3 = 10 t/ha (5.4 kg); FWC, *T*4 = 15 t/ha (8.1 kg); FWC, *T*5 = 5 t/ha (2.7 kg); LYC, *T*6 = 10 t/ha (5.4 kg); and LYC, *T*7 = 15 t/ha (8.1 kg) LYC treatment.

### 2.5. Soil and Compost Analysis

Before the application of compost treatment, one kilogram of composite soil samples was collected from the top 20 cm depth. The collected sample was separated from tree roots, leaves, and other unwanted materials and then air-dried and prepared for analysis of physical and chemical properties. Moreover, before the application of compost treatments, different compost samples were taken from different layers to determine the following selected physical and chemical properties: pH was determined in H_2_O (soil-H_2_O) at a 1 : 2.5 soil/compost-to-solution ratio using a pH meter, as described by Carter and Gregorich [24]. Electrical conductivity was measured using a conductivity meter after saturating the samples with distilled water and extracting them by vacuum suction, and the extracts were filtered [25]. Organic carbon was used to determine this using the Walkley and Black wet oxidation method [26]. Total N of the soil and compost was determined by Olsen et al. [27] using the micro-Kjeldahl method, digestion, distillation, and titration procedures. The Olsen method was used to determine the available phosphorous and potassium using sodium bicarbonate (0.5 M NaHCO_3_) as the extraction solution [27]. The ammonium acetate (1 M NH_4_OAc of pH 7) extraction method was used to determine the exchangeable bases (Ca^2+^ and Mg^2+^) in soil. In this procedure, Ca^2+^ and Mg^2+^ in the extracts were determined using an atomic absorption spectrophotometer, while the contents of exchangeable K were determined using a flame photometer, as described by Rowell [28]. The cation exchange capacity (CEC) of soil and compost was determined after leaching ammonium acetate-extracted (ammonium ion standard) soil samples with a 10% sodium chloride solution. The hydrometer method was used to determine the soil texture.

After the second harvesting period, soil samples were collected from each treatment plot to analyze the physical and chemical properties of soil, such as pH, electrical conductivity (EC), soil organic carbon (OC), total nitrogen (TN), available phosphorous, available potassium, exchangeable bases (Ca^2+^ and Mg^2+^), and cation exchange capacity (CEC) by following the standard procedure.

### 2.6. Growth Parameters

To record the growth and yield parameters of Swiss chard plants, field data were collected 10 days after transplanting seedlings [29]. The first and second harvests were carried out at the first cut (35 days) and the second cut (65 days) after transplanting seedlings. During harvesting, all of the mature outer leaves were removed, leaving only three small inner leaves. Following the field data collection period, the leaf numbers were counted twice per week until the experiment ended. Before cutting mature leaves, their height was measured in centimeters from the base to the top of the longest leaf of each plant. Then, the harvested leaves were cleaned with tap water, dried with paper towels, and weighed to determine the fresh weight (FW). Finally, the leaves were dried in a ventilated oven at 70°C until a steady weight was obtained to determine the dry weight (DW).

### 2.7. Statistical Analyses

The experiment was subjected to analysis of variance in the randomized complete block design, and data were analyzed using the R program (version 4.11.2021). To determine the significant difference between treatment means, Fisher's range test at a 5% significance level (*p* < 0.05) was applied.

## 3. Results and Discussion

### 3.1. Status of Selected Soil Properties before Experiment

Table 1 shows the analytical results for selected soil properties. According to the soil survey manual [30] and Murphy [31], the soil textural class of the study area where the experiment was established in plant growth was clay loam with very low saline content. Based on the report by Debele [32] and Murphy [31], the soil had medium soil organic carbon content (SOC), medium total nitrogen (TN) percentage, and very low available phosphorus (avail. P), respectively. The soil reaction was moderately acidic as reported by Murphy [31]. According to the FAO [33] rating, the result also showed a medium level of cation exchange capacity (CEC), high potassium (K), high calcium (Ca^+2^), and high magnesium (Mg^+2^).

### 3.2. Characteristics of Compost

Table 2 shows the physical and chemical properties of food waste compost (FWC) and leaf and yard compost (LYC). FWC and LYC had pH values of 6.55 and 6.31, respectively.

According to Murphy [31] ratings, the FWC and LYC compost was in the range of slightly acidic soil reactions. In general, the pH range of the compost produced at the experimental site was within the suitable standard of best compost production (a pH range of 6–8) reported by Alexander [34] and Neina [35]. The electrical conductivity (EC) of FWC and LYC was registered to be 0.04 and 0.03 dS/cm, respectively (Table 2).

According to Murphy [31]'s report, the electrical conductivity of both the produced compost was very low. The observed compost EC values of FWC and LYC meet the standard quality of compost [31]. Thus, the compost produced in this study using biodegradable organic waste was suitable for agricultural crops.

The soil organic carbon (SOC) content of composted food waste and leaf and yard waste was 2.92% and 2.4%, respectively (Table 2). The results showed that the SOC content of FWC was slightly higher than that of LYC. Because of the presence of various composting materials in food waste, it is helpful to increase organic matter. Similar findings were reported by Gajalakshmi and Abbasi and Szilveszter et al. [36, 37], who found that the type of feedstock used to produce compost has a significant impact on organic carbon, soil health, and plant growth.

The percentage of total nitrogen (TN) content in the compost produced ranged from 1.4 to 1.7 percent (Table 2). The amount of TN detected in FWC was higher (1.70%) than in LYC (1.40%). According to the report by Debele [32], the total nitrogen content of both FWC and LYC was high. Especially, the highest percentage of TN in FWC might be the composition of food waste materials, which helps to increase the amount of total nitrogen available in the produced compost.

The observed available phosphorous values in FWC and LYC were 1.60 and 1.35 ppm, respectively (Table 2). This study's findings were consistent with those of the Neina [35]'s report on the range of available phosphorus. Table 2 also shows that CEC was 35.18 meq/100 g soils for FWC and 32.46 meq/100 g soils for LYC, respectively. According to Landon [38], soil's cation exchange capacity (CEC) in LYC was in the medium range and high in FWC.

### 3.3. Effects of Food Waste and Leaf and Yard Compost on the Agronomic Performance of Swiss Chard

Table 3 shows the plant height (PH), number of leaves (NL), leaf area (LA), and fresh weight (FW) produced by Swiss chard plants during the two considered harvesting times, respectively, completed at 35 (first harvest) and 65 (second harvest) days after transplanting seedlings.

#### 3.3.1. Plant Height

The measured plant height (PH) at the first and second harvesting times was significantly different between amendments (*p* < 0.001), application rates (*p* < 0.001), and the interaction of amendments and application rates (*p* < 0.05). Among amendments, FWC showed the highest PH value both at the first and second harvesting time (15.35 ± 3.23 and 10.65 ± 1.44, respectively), followed by the plants treated with LYC (on average, 14.19 ± 2.44 and 10.10 ± 1.30, respectively, at the first and second harvesting times), and the lowest value of PH was registered in the control treatment (10.37 ± 0.28 and 8.65 ± 0.13, respectively). The PH in each treatment was higher when 15 t/ha organic amendment was applied in the treatment plots than at other applicable rates. Considering the interaction effect, the maximum values of plant height (19.37 ± 0.62 and 12.45 ± 0.21, respectively, at the first and second harvesting times) were observed in 15 t/ha FWC, while the least value was observed in the control treatment (Table 3).

The highest plant height observed in 15 t/ha FWC might be due to more nutrients and growth hormones available in compost that could improve growth medium porosity, aeration, and water retention capacity and promote plant height's fast growth. The current study was supported by the authors in [39, 40], indicating that the application of organic fertilizer, particularly compost, improved plant heights. Similar findings were also explored by Berova et al. [41] and Radhakrishnan and Mahendran [42], which indicated that the application of organic fertilizer increased plant height.

#### 3.3.2. Leaf Number

Table 3 reports that the leaf number (LN) was significantly influenced by organic amendments (*p* < 0.001), application rates (*p* < 0.001), and interaction of amendments and application rates (*p* < 0.001) in the first harvesting time. The highest average value of LN was recorded on plants treated with FWC (17.34 ± 4.33), followed by LYC (15.07 ± 2.68), while the lowest value was recorded from the control treatment (12.65 ± 0.19). Moreover, adding organic amendments of FWC and LYC increased leaf numbers by 27.05% and 20.24%, respectively, in comparison to the control treatment. Considering the application rates, the maximum value of LN was observed in plants treated with 15 t/ha, followed by 10 t/ha, while the minimum value was registered in the control and 5 t/ha treatments. The interaction effect of amendments and application rates indicated that the highest value of LN was observed with soil treated with 15 t/ha FWC (22.78 ± 2.32). Furthermore, plants treated with 15 t/ha FWC and 15 t/ha LYC increased LN by 44.47% and 31.84%, respectively, compared to the control treatment. At the second harvesting time, LN significantly differed among organic amendments (*p* < 0.05) and application rate (*p* < 0.001) (Table 3). The average LN value was higher in FWC and LYC (14.45 ± 0.70, and 13.47 ± 0.42, respectively) than in the control.

LN results were generally higher when the plants were treated with an application rate of 15 t/ha, followed by 10 t/ha, and the least was observed in the control treatment. Furthermore, the LN value was higher when 15 t/ha organic amendment was applied in the treatment plots than at other application rates. This finding was consistent with those by Masarirambi et al. [43], who reported that the application of organic fertilizer made from chicken manure increased the leaf numbers of lettuce plants more than the control. Eltun et al. [44] reported that the application of organic fertilizer might have increased the number of leaves on different plants. A similar result was also reported by the authors in [45, 46], who depicted that the application of organic fertilizer increased the number of leaves.

#### 3.3.3. Leaf Area

The leaf area (LA) value (Table 3) was always statistically different among the organic amendments (*p* < 0.001), application rate (*p* < 0.001), and the interaction effect (*p* < 0.05) in the first harvesting time. The soil treated with FWC and LYC had significantly higher values of LA than that with other treatments. On average, adding amendments of FWC and LYC increased LA by (30.32%) in FWC and 23.71% in LYC, respectively, compared to the control treatment. The interaction effect of amendments and application rates indicated that the highest value of LA was recorded with soil treated with 15 t/ha FWC (221.33 ± 4.04). In addition, plants treated with 15 t/ha FWC and 15 t/ha LYC increased LA by 42.32% and 34.53%, respectively, compared with the control treatment. At the second harvesting time, LA significantly differed among organic amendments (*p* < 0.05) and the application rate (*p* < 0.001) (Table 3). The average LA value was higher in FWC and LYC (143.67 ± 9.61, and 138.33 ± 6.51, respectively) than in the control. LN results were generally higher when the plants were treated with an application rate of 15 t/ha, followed by 10 t/ha, and the least was observed in the control treatment.

The study's findings were in agreement with those of the report of Bharadwaj and Nainawat [47], who mentioned that organic fertilizer increased the leaf area of two wheat varieties compared to the control. Similar findings reported by Xu et al. [48] revealed that the application of compost resulted in higher vegetative growth in the leaf area than in the control.

#### 3.3.4. Fresh Weight (g)

The fresh weight of the Swiss chard plant was significantly influenced by organic amendments (*p* < 0.001), application rates (*p* < 0.001), and their interaction (*p* < 0.001), both at the first and second harvesting times (Table 3). At the first and second harvesting times, the highest average fresh weight values were registered in plants treated with FWC (10.38 ± 2.08 and 6.80 ± 1.45), while the lowest value was observed in the control treatments (6.50 ± 0.16 and 4.84 ± 0.06). Moreover, plants treated with FWC increased fresh weight by 37.38% and 28.8%, respectively, over the control in the first and second harvesting times. The application rate and interaction effect of organic amendments also caused statistically significant differences in fresh weight in the first harvest compared with the second harvest. Surprisingly, the fresh biomass weight in 15 t/ha of FWC was 49 and 43% greater than in the control treatment in the first and second harvesting times, respectively (Table 3). This result was in agreement with the findings of Liu et al. [49] and Adhikari et al. [50], who reported that the application of organic fertilizers contributes to increasing the fresh biomass weight of crops. Furthermore, Ermias and Fanuel [39] observed that the highest rate of organic amendment application increased crop fresh biomass weight.

### 3.4. Effects of Food Waste and Leaf and Yard Compost on Selected Soil Properties

Table 4 shows the effect of FWC and LYC on selected soil properties (pH, OC, TN, available P, CEC, and soil exchangeable acidity) after the second harvesting of Swiss chard plants.

Soil pH was significantly influenced by organic amendments (*p* < 0.001), application rates (*p* < 0.001), and their interaction (*p* < 0.001) after harvesting the Swiss chard crop (Table 4). The results of the analysis indicated that the highest soil pH (6.07 ± 0.19) was observed from FWC, followed by LYC (5.98 ± 0.19), while the lowest soil pH (5.60 ± 0.02) was recorded from the control treatment. The soil pH in each treatment was higher when 15 t/ha organic amendment was applied in the treatment plots than at other application rates. In the interaction effect of amendments and application rates as a whole, the lowest soil pH value was recorded on soil treated with the control treatment and with the highest value of 6.23 ± 0.03 in 15 t/ha FWC, respectively. On the other hand, the highest rate of FWC improved soil pH by 10.1% when compared to initial soil pH (Tables 1 and 4). This finding was consistent with the findings of Mkhabela and Warman [51], who found that the presence of K+, Ca^2+^, Mg^2+^, and CEC in compost helped to increase the pH value of treated soil compared to the control. Similarly, Zingore et al. [52] reported that the release of cations and anions following manure mineralization affects the nutrient balance of the soil solution and, as a result, its reaction. The cation exchange capacity can increase soil pH by increasing potential cations and base saturation. After application of FWC, the mean value of soil pH also changed from moderately acidic to slightly acidic (Tables 1 and 4).

SOC was significantly influenced by organic amendments (*p* < 0.01), application rates (*p* < 0.001), and interaction effects (*p* < 0.001) after harvesting the Swiss chard crop (Table 3). The soil analysis result indicated that the highest SOC content was observed in soil treated with FWC, followed by LYC. Lowest SOC was recorded for the control treatment. Moreover, the highest application rate of FWC improved the soil organic carbon content of treated soils by 30.4% (from 1.58 to 2.27) (Tables 1 and 3). The findings of Hartl and Erhart [53] indicated that the application of organic amendments increased the SOC of treated soil more than that of the control. Furthermore, Frimpong et al. [54] and Trupiano et al. [55] assured that SOC contents were higher in soils treated with organic fertilizer than in the control. Similarly, Brown and Cotton [56] reported that soil organic carbon increased three-fold and microbial activities in the soil doubled when compost was applied to farmed land.

Total soil N was significantly influenced by organic amendments (*p* < 0.001), application rates (*p* < 0.001), and interaction of amendment and application rates (*p* < 0.001) after harvesting the Swiss chard crop. The highest value of soil total N was observed in plants treated with FWC amendments (0.164 ± 0.03), followed by LYC (0.153 ± 0.01), while the lowest value was recorded from the control treatment (0.10 ± 0.01). In view of the interaction effect of amendments and application rates, the maximum value of soil total N was observed in plants treated with 15 t/ha FWC (0.197 ± 0.01), followed by 15 t/ha LYC (0.161 ± 0.01), while the minimum value was recorded in the control (0.10 ± 0.01). Furthermore, plants treated with 15 t/ha FWC and 15 t/ha LYC increased soil total N by 49% and 38%, respectively, compared to the control treatment (Table 4) [55]. It was observed that the application of organic amendments to soil increased soil nitrogen content, subsequently improving soil quality. Various studies have found that organic amendments increase soil nitrogen content [13, 57, 58]. They found that the soil treated with municipal solid waste and manure increased total nitrogen by 60% and 40%, respectively, when compared to untreated soil. Similarly, the application of food waste compost at a rate of 15 t/ha increased the nitrogen level of the soil when compared to other treatments [59].

Available phosphorus in soil was significantly affected by amendments (*p* < 0.001), application rates (*p* < 0.001), and interaction of amendments and application rates (*p* < 0.001). The maximum value of soil available P was registered from plots treated with FWC (3.64 ± 0.42), followed by LYC (3.32 ± 0.55), and the lowest value (2.21 ± 0.06) was observed in the control treatment. Available P in the soil was higher when 15 t/ha organic amendment was applied in the treatment plots than at other application rates. Moreover, the interaction effect of amendment and application rates was recorded from a minimum value of 2.21 ± 0.06 in the control treatment and to a maximum value of 4.13 ± 0.02 in 15 t/ha FWC, respectively. The type of feedstock used for compost production may account for the increase in available P in 15 t/ha FWC. The result findings were consistent with those of [46, 60], who found that increasing the rate of organic amendment application linearly increased soil available. P. Zahrim et al. [61] also demonstrated that composting food waste increases soil N, P, and K values, creating a suitable soil environment for plant growth. Food waste compost was found to have higher mean values of available P than leaf and yard compost due to its nutrient-rich content. A similar finding reported by Zahrim et al. [61] indicated that the high application rate of municipal solid waste compost remarkably increased available P.

Soil CEC was significantly (*p* < 0.001) influenced by organic amendments (*p* < 0.001), application rates (*p* < 0.001), and interaction of amendments and application rates (*p* < 0.01). The application of FWC and LYC amendments significantly increased the cation exchange capacity (Table 4) of the treated soils. The soil with a 15 t/ha FWC application had the highest CEC value (32.55 ± 1.89), while the control treatment had the lowest CEC value (20.33 ± 0.05). It was also observed from the experimental result that the highest application of FWC improved soil CEC by 29.8% (from 22.85 to 32.55 cmol (+)/kg), while the control treatment depleted by 11% (from 22.85 to 20.33 cmol (+)/kg) when compared to the initial total soil CEC content (Tables 1 and 4). The highest mean values of CEC in 15 t/ha FWC could be attributed to the increased availability of basic cations accompanied by an increase in the soil organic carbon content due to the highest application rate of FWC (Table 4). Moreover, the availability of macronutrients might be attributed to the increase in the CEC value in 15 t/ha FWC. The findings of Meena et al. [62] assured that the high application rate of compost increases the CEC value of treated soil. Similarly, various findings indicated that the compost amendment increases CEC due to input from stabilized OM rich in functional groups such as carboxylic and phenolic acid groups being released into soil exchange sites as reported by Duong et al. [63]. In a study conducted by Li. et al. [64], compost treatments increased nutrient, organic carbon, and cation exchange capacity. Recently, the application of solid waste compost to clay loam soil has shown a variety of positive effects on soil chemical properties. Aside from increasing the OM content, it also increases the CEC and nutrient content of the soils [65].

Exchangeable acidity was also significantly (*p* < 0.001) affected by organic amendments, application rates (*p* < 0.001), and interaction effects (*p* < 0.001) (Table 4). The result of the analysis indicates that the highest exchangeable acidity (0.534 ± 0.01) was recorded from treatment supplied with the control, while the lowest exchangeable acidity (0.180 ± 0.01) was recorded from treatment supplied with 15 t/ha FWC (Table 4). The highest application rate of FWC reduced the soil exchangeable acidity value by 64% (from 0.535 to 0.180). Because of the impact of compost on soil OC decomposition, the reduction in soil exchangeable acidity for FWC applied at 15 t/ha is most likely due to the release of basic cations into the soil solution, which may hydrolyze and react with soluble Al^+3^ ions to form insoluble Al(OH)_3_ and water. On the other hand, when the exchangeable acidity of the soil is high, resulting in low pH, it affects the soil condition and many soil processes. Furthermore, the bioavailability of iron, aluminum, or manganese could be very high, reaching toxic levels at lower pH [49].

## 4. Conclusion

This research was conducted with the purpose of investigating the effects of different rates of FWC and LYC on selected soil properties and the yield of a Swiss chard crop under irrigation. Field experimental results showed that Swiss chard responded positively to the application of organic amendments. Particularly when the maximum application rate of FWC is added to the soil, it significantly increases soil pH by 10%, SOC by 30%, TN by 49%, AP by 47%, and CEC by 39% and reduces soil exchangeable acidity by 64% compared to the control. Moreover, the highest application rate of FWC improved the plant height, leaf number, leaf area, and fresh weight of Swiss chard when compared with the control treatment. Therefore, based on the present study findings, the application of 15 t/ha FWC could be recommended as a potential fertilizer to improve soil properties and yields of Swiss chard.

## Figures and Tables

**Table 1:** Selected properties of soil.

Parameters	Unit	Value	Rating	Source
pH (H_2_O)		5.6	Moderately acidic	Murphy [31]
EC	dS/cm	0.04		Murphy [31]
SOC	%	1.58	Medium	Debele [32], Murphy [31]
Avail. P	ppm	3.11	Low	Debele [32], Olsen et al. [27], Murphy [31]
TN	%	0.106	Medium	Debele [32], Murphy [31]
Na	meq/100 g	1.186		FAO [33], Murphy [31]
K	meq/100 g	1.22	High	FAO [33], Murphy [31]
Ca^2+^	meq/100 g	11.33	High	FAO [33]
Mg^2+^	meq/100 g	6.02	High	FAO [33]
CEC	cmol (+)/kg	22.85	Medium	Murphy [31]
Exchangeable acidity	meq 100 g−1	0.508	—	Murphy [31]
Textural class			Clay loam	U.S. Department of Agriculture [30], Murphy [31]

Source: soil laboratory analysis result report.

**Table 2:** Physical and chemical properties of compost.

Parameters	FWC	LYC
pH (1 : 2.5 H_2_O)	6.55	6.31
EC (dS/cm)	0.04	0.03
% OC	2.92	2.41
Avail. P (ppm)	1.60	1.35
%TN	1.70	1.40
K (meq/100 g)	1.45	1.30
Ca (meq/100 g)	16.73	14.91
Mg (meq/100 g)	9.17	7.88
CEC (cmol (+)/kg)	35.18	32.46

**Table 3:** The effect of organic amendments on growth performance of Swiss chard.

Treatment (T)	Application rate (AR)	First harvest				Second harvest			
		PH (cm)	LN	LA (cm^2^)	FW (g)	PH (cm)	LN	LA (cm^2^)	FW (g)
Control	No	10.37 ± 0.28^g^	12.65 ± 0.19^e^	127.67 ± 2.52^d^	6.53 ± 0.21^i^	8.65 ± 0.13^g^	8.14 ± 0.13^d^	91.00 ± 3.61^f^	4.84 ± 0.06^de^

FWC	5	12.19 ± 0.19^e^	13.67 ± 0.42^d^	147.33 ± 7.02^c^	8.18 ± 0.20^e^	9.16 ± 0.03^d^	9.19 ± 0.61^d^	118.33 ± 3.06^d^	5.14 ± 0.04^d^
	10	14.49 ± 0.99^c^	15.55 ± 0.17^c^	181.00 ± 6.56^b^	10.06 ± 0.17^c^	10.35 ± 0.12^c^	11.97 ± 0.30^c^	129.00 ± 3.61^b^	6.80 ± 0.19^c^
	15	19.37 ± 0.62^a^	22.78 ± 2.32^a^	221.33 ± 4.04^a^	12.90 ± 0.56^a^	12.45 ± 0.21^a^	14.45 ± 0.70^a^	143.67 ± 9.61^a^	8.46 ± 0.23^a^

LYC	5	11.73 ± 0.38^f^	12.76 ± 0.45^e^	142.33 ± 2.52^c^	7.25 ± 0.13^f^	8.58 ± 0.12^e^	8.92 ± 0.39^d^	112.00 ± 2.00^e^	5.02 ± 0.11^d^
	10	13.62 ± 0.28^d^	13.88 ± 0.25^d^	164.67 ± 5.03^bc^	9.33 ± 0.14^d^	10.14 ± 0.16^c^	11.35 ± 0.31^c^	122.00 ± 2.00^c^	6.17 ± 0.17^c^
	15	17.23 ± 0.43^b^	16.84 ± 0.13^b^	195.00 ± 12.12^b^	11.80 ± 0.32^b^	11.58 ± 0.14^b^	13.47 ± 0.42^a^	138.33 ± 6.51^a^	7.47 ± 0.37^b^

T		^*∗∗∗*^	^*∗∗∗*^	^*∗∗∗*^	^*∗∗∗*^	^*∗∗∗*^	^*∗*^	^*∗*^	^*∗∗∗*^
AR		^*∗∗∗*^	^*∗∗∗*^	^*∗∗∗*^	^*∗∗∗*^	^*∗∗∗*^	^*∗∗∗*^	^*∗∗∗*^	^*∗∗∗*^
Tx AR		^*∗*^	^*∗∗∗*^	^*∗*^	Ns	^*∗*^	Ns	Ns	^*∗∗*^

FWC = food waste compost; LYC = leaf and yard compost. Values followed by similar letters under the same column are not significantly different. ^*∗∗∗*^*p* < 0.001. ^*∗∗*^*p* < 0.01. ^*∗*^*p* < 0.05.

**Table 4:** Effect of organic amendments on selected soil properties.

Treatment (T)	Application rate (AR)	pH	EC (dS/cm)	SOC (%)	TN (%)	Ava. P	CEC	Exc. acidity
Control	No	5.60 ± 0.02^e^	0.070 ± 0.002^a^	1.56 ± 0.02^f^	0.102 ± 0.002^g^	2.21 ± 0.06^g^	20.33 ± 0.005^f^	0.534 ± 0.003^a^

FWC	5	5.82 ± 0.04^d^	0.066 ± 0.001^b^	1.60 ± 0.01^e^	0.122 ± 0.002^e^	3.17 ± 0.03^e^	22.44 ± 0.03^e^	0.487 ± 0.002^b^
	10	6.15 ± 0.03^b^	0.031 ± 0.003^d^	1.86 ± 0.02^c^	0.136 ± 0.004^c^	3.60 ± 0.06^c^	27.01 ± 0.03^c^	0.312 ± 0.003^d^
	15	6.23 ± 0.03^a^	0.013 ± 0.002^f^	2.27 ± 4.02^a^	0.164 ± 0.002^a^	4.13 ± 0.02^a^	32.55 ± 1.89^a^	0.180 ± 0.002^g^

LYC	5	5.80 ± 0.06^d^	0.055 ± 0.001^c^	1.69 ± 0.02^d^	0.115 ± 0.005^f^	2.64 ± 0.08^f^	24.40 ± 0.08^d^	0.393 ± 0.002^c^
	10	5.92 ± 0.03^c^	0.034 ± 0.003^d^	1.85 ± 0.02^c^	0.132 ± 0.002^d^	3.42 ± 0.03^d^	26.85 ± 0.22^c^	0.337 ± 0.006^e^
	15	6.21 ± 0.03^a^	0.028 ± 0.004^e^	2.11 ± 0.02^b^	0.153 ± 0.002^b^	3.91 ± 0.07^b^	30.61 ± 0.67^b^	0.224 ± 0.002^f^

T		^*∗∗∗*^	^*∗*^	^*∗∗*^	^*∗∗∗*^	^*∗∗∗*^	^*∗∗*^	^*∗∗∗*^
AR		^*∗∗∗*^	^*∗∗∗*^	^*∗∗∗*^	^*∗∗∗*^	^*∗∗∗*^	^*∗∗∗*^	^*∗∗∗*^
Tx AR		^*∗∗∗*^	^*∗∗∗*^	^*∗∗∗*^	^*∗∗∗*^	^*∗∗∗*^	^*∗∗*^	^*∗∗∗*^

FWC = food waste compost; LYC = leaf and yard compost. Values followed by similar letters under the same column are not significantly different. ^*∗∗∗*^*p* < 0.001. ^*∗∗*^*p* < 0.01. ^*∗*^*p* < 0.05.

## Data Availability

The raw data that support the findings of this study are available upon request from the corresponding author based on reasonable request.
